# An Unexpected Cause of Persistent Coughing

**DOI:** 10.5811/cpcem.2018.2.37443

**Published:** 2018-04-05

**Authors:** Melissa Myers, Jared Cohen

**Affiliations:** San Antonio Military Medical Center, Department of Emergency Medicine, San Antonio, Texas

## CASE PRESENTATION

**Image 1:**
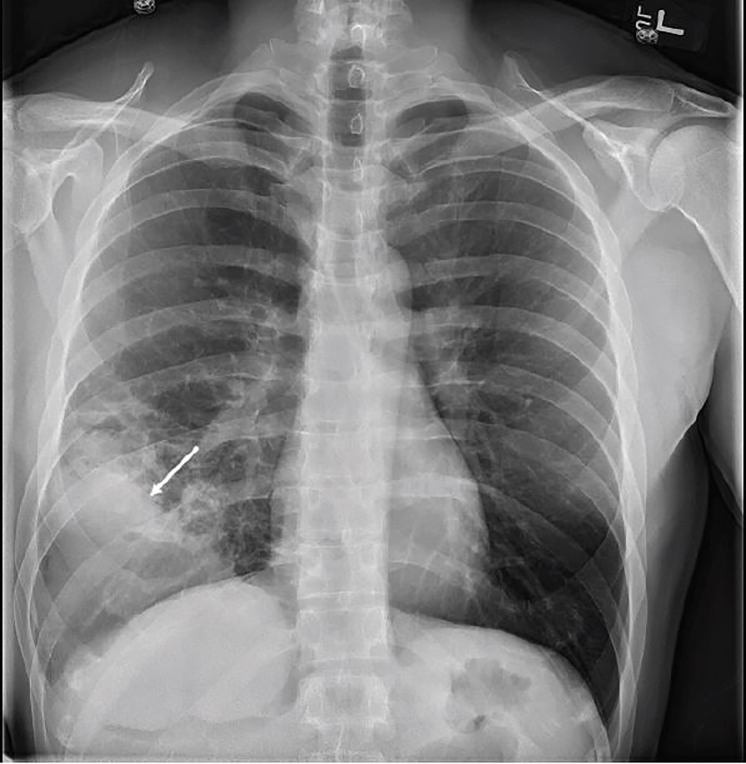
Chest radiograph demonstrating a consolidation (arrow).

**Image 2:**
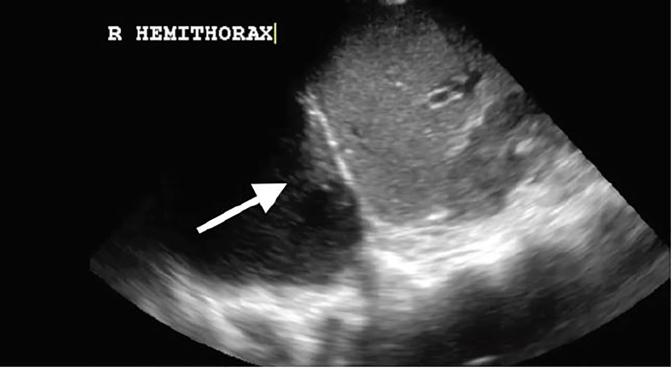
Thoracic point-of-care ultrasound using 4-megahertz curvilinear probe, demonstrating an empyema in a coronal view (arrow). See video.

**Image 3:**
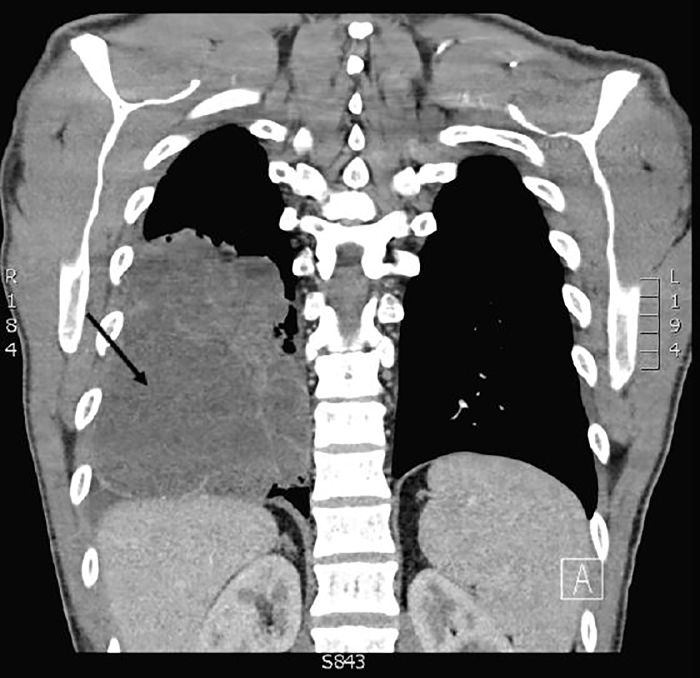
Chest computed tomography demonstrating a right-sided pulmonary abscess (arrow).

A 35-year-old, otherwise-healthy male presented to a military emergency department complaining of persistent cough. He had been treated for community-acquired pneumonia three weeks prior to this presentation with a five-day course of azithromycin. His vital signs were stable with no fever, hypoxemia or respiratory distress. His physical exam was unremarkable with no focal lung findings. Chest radiograph (Image 1) was performed and was concerning for consolidation or effusion. Pulmonary point-of-care ultrasound (POCUS) was then performed with results seen in Image 2 and video. POCUS showed a loculated effusion consistent with empyema. Chest computed tomography (Image 3) showed a large, lung abscess. The patient was admitted to the hospital and underwent video-assisted thoracoscopic surgery for evacuation of the lung abscess caused by pan-sensitive *streptococcus pneumoniae*. He ultimately recovered and was discharged without complications.

## DIAGNOSIS

Pulmonary POCUS can be used at the bedside to diagnose and describe pleural effusions. The exam is performed using a low-frequency probe placed in the posterior axillary line in a longitudinal view. Fluid collections will be visible directly above the diaphragm. Transudates will appear anechoic and simple on ultrasound. A more complex appearance with complex septations or heterogenous appearance indicates the presence of an exudate.^1^ Empyema may be further distinguished by a “snow flurry” or “Swiss cheese” appearance.^2^ Sensitivity of lung POCUS for pleural effusion is greater than 95%, compared to a 65% sensitivity for chest radiography.^3^ POCUS offers a rapid, sensitive method to evaluate for pulmonary pathology. Combining ultrasound findings with history and physical exam can increase physician sensitivity for common diagnoses and improve early diagnosis and treatment.

**CPC-EM Capsule**

What do we already know about this clinical entity?

An empyema is a complication of pneumonia where the pleural cavity is filled with a purulent effusion.

What is the major impact of the image(s)?

Pulmonary point-of-care ultrasound can improve the early diagnosis and differentiation of pleural effusions when compared to chest radiography.

How might this improve emergency medicine practice?

Incorporating point-of-care ultrasound early in the management of patients with abnormal lung findings may improve care by improving diagnostic accuracy.

Documented patient informed consent and/or Institutional Review Board approval has been obtained and filed for publication of this case report.

## Supplementary Information

Video Thoracic point-of-care ultrasound using 4-megahertz curvilinear probe, demonstrating an empyema in a coronal view (arrow).
